# Genetic analysis of three familial cases of pure terminal 19p13.3 duplication caused by maternal balanced translocation t(19;21) (p13.3;p12)

**DOI:** 10.3389/fgene.2026.1827876

**Published:** 2026-06-02

**Authors:** Jia-yan Chen, Mei-jiao Cai, Xiao-lu Chen, Yun-sheng Ge

**Affiliations:** Department of Central Laboratory, Women and Children’s Hospital, School of Medicine, Xiamen University, Xiamen, China

**Keywords:** 19p13.3 microduplication syndrome, CELF5, developmental delay, DOHH, FZR1, intrauterine growth restriction, microcephaly, NFIC

## Abstract

**Introduction:**

This study presents a genetic analysis of three-generation family exhibiting terminal 19p13.3 duplication resulting from a maternal balanced translocation, t (19; 21) (p13.3; p12). Additionally, we reviewed previously reported cases with similar aberrations.

**Methods:**

Peripheral blood or amniotic fluid samples from six family members were comprehensively analyzed using G-banding, N-banding, fluorescence in situ hybridization (FISH), and copy number variation sequencing (CNV-seq). Furthermore, a genomic mapping analysis was performed on 23 cases of terminal 19p13.3 duplication, combining our findings with those from previously reported cases.

**Results:**

The results demonstrated that the maternal balanced translocation resulted in three offspring inheriting the 19p13.3 terminal duplication. The proband presented with typical clinical features such as intrauterine growth restriction, microcephaly, intellectual disability, developmental delay, and facial abnormalities, in addition to precocious puberty, autism spectrum disorder (ASD) features, and a shortened lingual frenulum. The precocious puberty phenotype is postulated to be associated with the KISS1R gene within the duplicated region. Genomic mapping identified a minimal overlapping region (MOR) of approximately 313 kb (chr19:3,223,850–3,536,224), encompassing four OMIM genes (CELF5, NFIC, DOHH, FZR1) and two protein-coding genes (SMIM24, SMIM44).

**Conclusion:**

This study clarifies the genetic mechanism of pure terminal 19p13.3 duplication in offspring resulting from a parental balanced translocation involving D/G group chromosomes. It also defines a novel critical region based on case samples with 19p13.3 terminal duplication, providing new insights into the genotype-phenotype correlations associated with terminal pure 19p13.3 duplications.

## Introduction

Among all human chromosomes, chromosome 19 exhibits the highest gene density, more than double the genomic average (Jouret et al., 2022; Orellana et al., 2015; Tenorio et al., 2020). However, relatively few disease-associated microdeletion and microduplication regions have been characterized to date. On the short arm, the 19p13.3 locus has been implicated in microdeletion and microduplication syndromes. This segment spans approximately 6.9 Mb and contains about 307 genes (Özer et al., 2021). Among abnormalities associated with this region, microdeletions predominate, whereas microduplications are exceedingly rare and frequently co-occur with deletions on other chromosomes (Bain et al., 2023). Isolated cases of pure terminal duplication of 19p13.3 are exceedingly rare, with the first instance reported in 2002 (Andries et al., 2002). To date, only 20 such cases have been documented. The clinical phenotypes of these microduplication cases are highly similar, including intrauterine growth restriction, microcephaly, intellectual disability, language and developmental delay, and distinctive facial abnormalities (prominent forehead, low-set ears, micrognathia), collectively termed 19p13.3 microduplication syndrome (Siggberg et al., 2011; Ishikawa et al., 2013; Novikova et al., 2017). This study reports a family in which the mother carries a balanced t (19; 21) (p13.3; p12) translocation, resulting in terminal pure duplication of 19p13.3 in three offspring. To our knowledge, this constitutes the first reported family with terminal pure duplication of 19p13.3 arising from a parental balanced translocation involving the short arms of chromosomes D and G. Additionally, this paper reviews 20 previously reported cases of isolated terminal pure duplication of 19p13.3 to enhance clinicians’ understanding of this condition.

## Clinical history

Patient 1, an 8-year-old female, was born at term via vaginal delivery as the second child of healthy, non-consanguineous parents with an unremarkable family history. Due to intrauterine growth restriction (IUGR), her birth weight was only 1.85 kg, and microcephaly was noted at birth. At 13 months, she was referred to our hospital for motor delays, specifically an inability to crawl or stand with support, and subsequently underwent three courses of rehabilitation therapy. Subsequent growth and development remained delayed: at 19 months, her weight was 7.5 kg and head circumference was 40 cm (<−3 SD); she could stand with support and walk while holding hands, but could not stand or walk independently. The ability to grasp and transfer toys with both hands was present, but a pincer grasp was absent. Responses to sounds were observed, but reactions to the surroundings were weak; she remained non-verbal and could not produce words such as “Dad” or “Mom”. Muscle tone was normal and cranial MRI revealed no significant abnormalities. Blood amino acid, acylcarnitine, and urinary organic acid metabolic screening were unremarkable. EEG was normal; brainstem auditory evoked potentials (BAEP) demonstrated poor wave differentiation; Neuropsychological Developmental Scale assessment (0–6 years) indicated global developmental delay (GDD) (DQ = 66.9, Gross Motor 71, Fine Motor 63.5, Adaptive Skills 69.1, Language 67.3, Social Behavior 63.5).

At 24 months, independent walking was achieved; at 27 months, verbal expression remained absent, signalling for toileting needs was not possible, social interaction was poor, and stereotyped behaviors and narrow interests were present; head circumference was 41 cm (<−3SD). At 40 months, spontaneous speech was still lacking, brief eye contact could be made, actions could be imitated, and head circumference was 43 cm (<−3SD). Facial features included micrognathia, hypertelorism (wide-set eyes), and strabismus, in addition to a shortened lingual frenulum. A diagnosis of Global Developmental Delay (GDD) with autism spectrum disorder (ASD) features and moderate intellectual disability was made.

At age 6 years and 7 months, head circumference was 44 cm (<−3SD), facial features persisted, and bilateral breast enlargement persisting for 6 months (Tanner stage: bilateral stage BII) with vulvar stage PH I prompted consideration of potential precocious puberty.

Prenatal diagnosis was not performed for the proband’s mother during this pregnancy. Following the proband’s birth, peripheral blood chromosome karyotyping and copy number variation sequencing (CNV-seq) analysis were performed due to GDD. Both tests indicated abnormalities. Subsequently, the same experiments were performed on the parents for verification. Genetic verification confirmed that the chromosomal abnormality in the proband was inherited from the mother, who was identified as a carrier of a balanced translocation. The mother has a history of five pregnancies: the first pregnancy was terminated at 25 weeks due to “oligohydramnios and fetal growth restriction,” without genetic testing. The second pregnancy resulted in the proband. Subsequently, the mother had three additional pregnancies, all of which underwent amniocentesis for prenatal diagnosis between 16 and 17 weeks gestation.

## Study materials

Peripheral blood samples were collected from the proband and her parents. Amniotic fluid samples from the mother’s third, fourth, and fifth pregnancies were obtained via amniocentesis at gestational ages of 16^+2^, 17, and 16^+3^ weeks, respectively. Chromosomal karyotyping and CNV-seq analysis were performed on all samples. Where necessary, N-banding and FISH techniques were employed for validation.

## Research methods

1. Chromosome G-banding Karyotyping: The collected peripheral blood and amniotic fluid specimens were subjected to standard culturing, harvesting, and slide preparation procedures. For each case, 20–100 well-dispersed metaphase divisions were counted, and at least five cells were fully analyzed. Karyotypes were described according to the International System of Nomenclature for Human Cytogenetics (ISCN 2024).
2. CNV-seq Analysis: Genomic DNA was fragmented and sequenced using the Illumina HiSeq2000 platform with an average sequencing depth of 0.1×. Sequence reads were subsequently aligned to the human reference genome (hg19/GRCh37) using the Burrows-Wheeler algorithm.
3. N-banding Analysis: Prepared chromosome slides underwent N-banding according to standard operating procedures, followed by examination and analysis using a phase-contrast microscope.
4. FISH Analysis: Following standard FISH protocols, commercial probes for 19pter, 19qter, and 21qter were hybridized to prepared metaphase spread. Signals were then observed and analyzed under a fluorescence microscope.
5. Genomic Mapping: An integrated analysis was performed on 23 cases of terminal 19p13.3 duplication (including 20 previously reported cases and 3 novel cases identified in this study), 22 of which had clear genomic coordinates for the duplicated segments. The duplicated regions in all cases were mapped to the genome using the UCSC Genome Browser. Subsequently, the minimal overlapping region (MOR) common to all cases was identified by comparing their respective intervals.

## Experimental results and pregnancy outcome

1. Maternal Genetic Evaluation.

**FIGURE 1:**
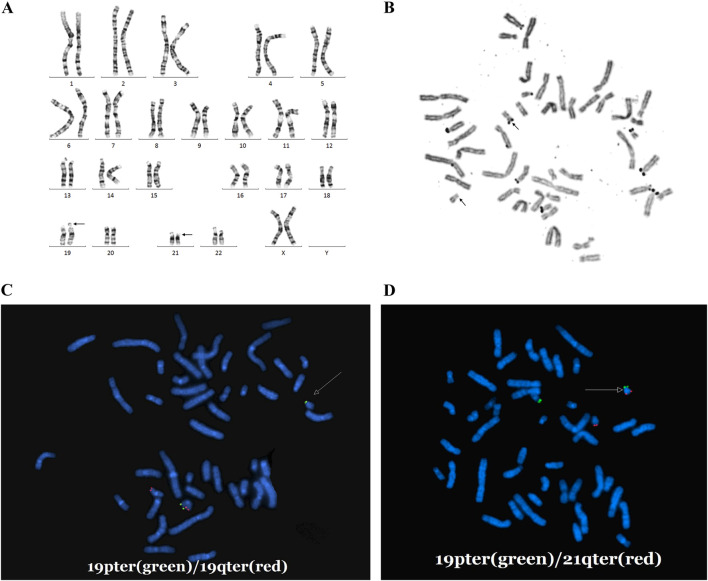
Cytogenetic analysis of the proband’s mother. **(A)** Chromosome G-banding karyotype. The arrow highlights a balanced translocation between the terminal short arm of chromosome 19 and the satellite region of chromosome 21. **(B)** Chromosome N-banding result. The arrow demonstrates the translocation of the chromosome 21 satellite to the terminal end of chromosome 19. **(C)** Fluorescence *in situ* hybridization (FISH) using 19pter/19qter probes. The arrow indicates the 19pter signal localized to the terminal short arm of a D/G group chromosome. **(D)** FISH using 19pter/21qter probes. The arrow confirms the 19pter signal on the terminal short arm of chromosome 21.

CNV-seq analysis of the proband’s mother revealed no genomic imbalances. However, G-banded karyotyping of peripheral blood showed additional material, appearing as a satellite, on the distal short arm of chromosome 19, while the satellite region on chromosome 21 was replaced by a light-staining band (Figure 1A), suggesting a potential balanced t (19; 21) (p13.3; p12) translocation.

To validate this finding, N-banding and FISH analyses were performed on metaphase chromosomes from the mother’s peripheral blood. N-banding revealed distinct double black signals at 19qter, confirming the presence of a satellite structure at this location. Conversely no black signal was observed on chromosome 21qter, indicating loss of its satellite (Figure 1B). FISH analysis using 19pter, 19qter, and 21qter probes demonstrated that the 19pter probe signals were localized on chromosome 21 (Figures 1C,D).

Integrating these findings, the maternal karyotype was formally determined as: 46,XX,t (19; 21) (p13.3; p12), representing a balanced translocation between band p13.3 of chromosome 19 and the satellite region of chromosome 21.

**FIGURE 2:**
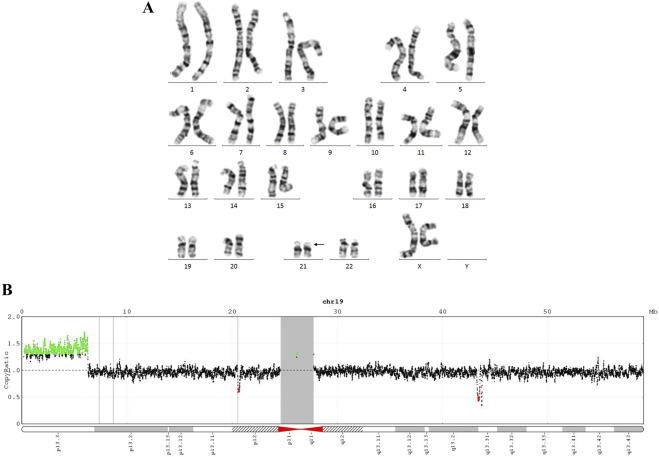
The diagnostic findings obtained from the proband. **(A)** The G-banded karyotype, in which it is elucidated that an abnormal chromosome 21-indicated by the arrow-was inherited maternally. **(B)** The results of copy number variation sequencing (CNV-seq), through which a terminal pure duplication spanning approximately 6.10 megabases within the 19p13.3 region (chr19:178,608–6,276,672) is identified in the proband. These observations collectively posit a genomic aberration of maternal origin with a precisely delineated structural duplication.

**FIGURE 3:**
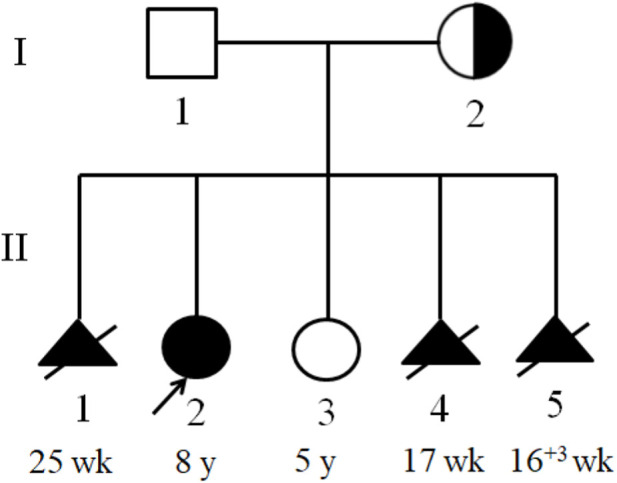
Genetic pedigree of the family. 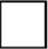: Normal male; 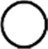: Normal female; 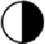: Carrier female; 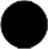: Affected female; 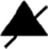: Affected fetus with termination of pregnancy; 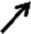: The arrow indicates the proband.

**FIGURE 4:**
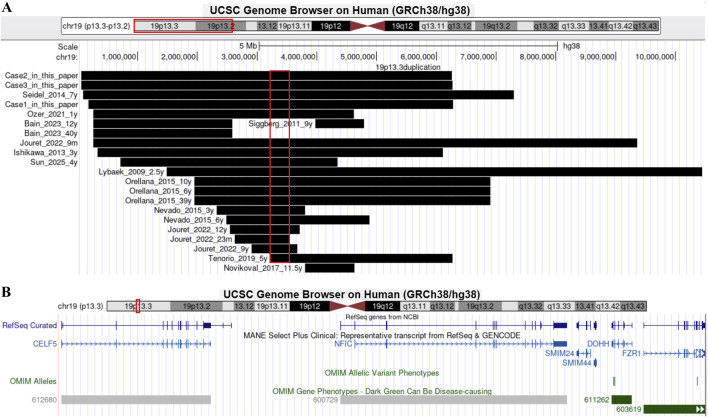
The genomic characteristics of 22 reported cases in pure 19p13.3 duplications. **(A)** The chromosomal locations and respective sizes of these duplications are depicted, with the smallest region of overlap (SRO)-spanning chr19:3,223,850–3,536,224-highlighted within a red box to emphasize the minimal shared duplicated interval. **(B)** This SRO encompasses four genes catalogued in the Online Mendelian Inheritance in Man (OMIM) database (CELF5, NFIC, DOHH, and FZR1), as well as two additional protein-coding genes (SMIM24 and SMIM44).

2. Paternal Evaluation: No abnormalities were detected in the proband’s father via karyotyping or CNV-seq.
3. Proband Evaluation: The proband inherited the derivative chromosome 21 from the mother, resulting in a partial 19p13.3 trisomy (Figure 2A). CNV-seq analysis revealed a terminal pure duplication segment of approximately 6.10 Mb at 19p13.3 (chr19:178,608–6,276,672) (Figure 2B). As the concurrent deletion involved only the heterochromatic satellite region of chromosome 21, it was not detectable by CNV-seq. The proband’s karyotype was confirmed as 46, XX, der (21) t (19; 21) (p13.3; p12) dmat.
4. Prenatal Diagnosis of Subsequent Pregnancies: In subsequent pregnancies of the proband’s mother, the third fetus inherited normal maternal chromosomes 19 and 21, with no abnormalities detected in karyotype analysis or CNV-seq testing. Currently, she is an asymptomatic 5-year-old girl. However, the fourth and fifth fetuses inherited the same derived chromosome as the proband. CNV-seq detected duplications of approximately 6.19 Mb (chr19:61,244–6,255,433) and 6.2 Mb (chr19:61,244–6,271,209) at 19p13.3, respectively. The karyotype for both was 46,U,der (21) t (19; 21) (p13.3; p12) dmat. Following genetic counseling, the parents opted for termination of pregnancies (Figure 3 for the genetic pedigree).
5. Genomic Mapping and Genotype–Phenotype Correlation: The duplicated regions from 22 cases of isolated terminal 19p13.3 duplications were mapped according to fragment size in Figure 4A. Integrated analysis revealed that 18 cases share a MOR (chr19:3,223,850–3,536,224). This region spans approximately 313 kb and encompasses four OMIM genes (CELF5, NFIC, DOHH, FZR1) and two protein-coding genes (SMIM24, SMIM44) (Figure 4B).

## Discussion

19p13.3 microduplication syndrome is a recently characterized intrauterine growth retardation syndrome (Seidel et al., 2014), which can be primarily classified into two categories: the first involves terminal 19p13.3 duplication associated with other chromosomal segment deletions, often arising from parental balanced translocations; the second is characterized by isolated terminal 19p13.3 pure duplication. To elucidate the association between the 19p13.3 duplication fragment *per se* and the clinical phenotype, statistical analysis was performed exclusively on cases of isolated terminal 19p13.3 pure duplication.

Such isolated cases are extremely rare; since the first report in 2002, only 20 cases have been documented in the literature (Jouret et al., 2022; Orellana et al., 2015; Tenorio et al., 2020; Özer et al., 2021; Bain et al., 2023; Andries et al., 2002; Siggberg et al., 2011; Ishikawa et al., 2013; Novikova et al., 2017; Seidel et al., 2014; Lybaek et al., 2009; Nevado et al., 2015; Sun et al., 2025). The clinical characteristics and molecular genetic of these 20 cases were summarized in Tables 1, 2. As shown in the tables, among the 16 patients with available gender information, the male-to-female ratio was 6:10. The age range was broad, spanning from 9 months to 40 years. The size of the duplicated segments ranged from 0.77 Mb to 9.1 Mb. Regarding the duplication type, all cases were homozygous duplicates except for one mosaicism reported by Ozer et al. Regarding genetic origin, 10 cases were *de novo* occurrences, 4 cases resulted from maternal chromosomal insertions of a 19p13.3 fragment into an autosomal long arm of chromosome 19, 1 case was associated with a maternal balanced translocation, and the inheritance mechanism for the remaining 5 cases was unspecified. Notably, only the mosaic patient reported by Ozer et al. demonstrated significant height improvement following growth hormone therapy; no similar outcomes were observed in the other cases.

**TABLE 1:** Molecular genetic characterization of individuals with pure 19p13.3 microduplication.

References	Andries et al. (2002)	Lybaek et al. (2009)	Siggberg et al. (2011)	Ishikawa et al. (2013)	Seidel et al. (2014)	Nevado et al. (2015)		Orellana et al. (2015)	Novikova et al. (2017)	Tenorio et al. (2019)
Reported cases	1	1	1	1	1	2		3	1	1
Start of first duplicated probe (hg19)	N/A	1,489,000	3,976,000	327,273	90,897	2,487,767	2,329,320	1,952,590	3,804,495	3,223,850
End of last duplicated probe (hg19)	N/A	10,449,000	4,790,000	6,106,229	7,300,043	4,882,351	3,808,325	6,908,729	4,633,722	6,267,526
Region size (Mb)	N/A	8.9	0.81	6.1	7.2	2.39	1.48	4.95	0.83	3.04
Number of genes within the 19p13.3 region	N/A	215	N/A	150	211	N/A	N/A	253	21	N/A
Mechanism underlying the 19p13.3 chromosomal segment duplication	N/A	46,XX,ins (19)(q13.3p13.2p13.3)dmat	*De novo*	46,XX,der (19)t (10; 19)(qter; p13.3)dn	46,XY,der (14)t (14; 19)(p11.2; p13.2)dn	*De novo*	*De novo*	A fragment of 19p13.3 was inserted into the 19q region in the maternal genome	N/A	N/A

References	Ozer et al. (2021)	Jouret et al. (2022)				Bain et al. (2023)	Sun et al. (2025)	Cases in this paper			Total
Reported cases	1	4				2	1	3			23
Start of first duplicated probe (hg19)	259,395	2,629,781	2,905,872	266,117	2,549,201	260,912	715,417	178,608	61,244	61,244	
End of last duplicated probe (hg19)	4,615,348	3,536,224	3,674,858	9,362,746	3,709,064	2,587,578	4,346,195	6,276,672	6,255,433	6,271,209	
Region size (Mb)	4.4 (mosaic duplication: 27.5% in blood lymphocytes and 47% in buccal epithelium)	0.91	0.77	9.1	1.16	2.3	3.6	6.1	6.19	6.21	
Number of genes within the 19p13.3 region	N/A	N/A	N/A	N/A	N/A	88	127	​	​	​	
Mechanism underlying the 19p13.3 chromosomal segment duplication	*De novo*	*De novo*	*De novo*	*De novo*	*De novo*	The grandmother was identified as a carrier of a balanced translocation t (15; 19)(q26.3; p13.3)	N/A	The grandmother was identified as a carrier of a balanced translocation t (19; 21)(p13.3; p12)			

**TABLE 2:** Summary of clinical characteristics in patients with pure 19p13.3 microduplication.

References	Andries et al. (2002)	Lybaek et al. (2009)	Siggberg et al. (2011)	Ishikawa et al. (2013)	Seidel et al. (2014)	Nevado et al. (2015)		Orellana et al. (2015)			Novikova et al. (2017)	Tenorio et al. (2019)
Reported cases	1	1	1	1	1	2		3			1	1
Age	21 m	2.5 years	9 years	3 years	7 years	6 years	3 years	10 years	6 years	39 years	11.5 years	5 years
Gender	M	F	M	F	M	F	F	M	M	F	M	F
Height	N/A	Birth height (35w): 42.5 cm (3%)	Birth height: 46 cm (−2.6 SD)	75 cm (−5 SD)	Birth height (32^+2^w): 34 cm (<3%)	N/A	Short stature	Birth height: 45 cm (<3%)	Birth height: 49 cm (<30%)	Birth height: 53 cm	132.5 cm (<3%)	Birth height: 43 cm (3%∼10%)
Weight	N/A	Birth weight (35w): 1.79 kg (3%)	Birth weight: 2,730 g (−1.9 SD)	7.68 kg (−3.4 SD)	Birth weight (32^+2^w): 990 g (<3%)	N/A	N/A	Birth weight: 1820 g (<3%)	Birth weight: 2.3 kg (<3%)	Birth weight: 2.18 kg (<3%)	26.8 kg (<10%)	Birth weight: 2.025 kg (<3%)
Intrauterine/growth disability	–	+	+	+	+	+	+	+	+	+	+	+
Psychomotor developmental delay/intellectual disability	+	+	+	+	+	+	+	+	+	+	+	+
Microcephaly	+ (−3 SD)	– (birth HC (35w): 30.5 cm (10%)	+ (birth HC: 32 cm (−2.2 SD)	+ (42 cm (−4 SD))	+ (birth HC (32^+2^w): 26.5 cm, <3%)	+ (−4.4 SD)	–	+ (birth HC: <3%)	+ (birth HC: 32.5 cm, <3%)	+ (44 cm at 22nd month)	+ (50.5 cm (5%))	+ (31 cm (10%))
Facial dysmorphism	+	+	NA	+	+	+	+	+	+	+	+	N/A
Other features	NA	Precocious puberty, left finger malformation, ventricular septal defect, congenital hip dysplasia, Severe feeding difficulties	Severe amblyopia of the right eye and hyperopia, epilepsy	Atrial septal defect, congenital heart disease, congenital hip dysplasia	Primary immunodeficiency disorder, congenital hip dysplasia, respiratory distress syndrome, bilateral incarcerated inguinal hernia, perineal hypospadias, urethral fistula, moderate bilateral sensorineural hearing loss, epilepsy, severe osteopenia	Digital abnormalities of the fingers and toes	Feeding difficulties, ophthalmologic abnormalities, heart disease	Cleft palate, pes cavus, clinodactyly, long fingers	Congenital hip dysplasia	Genu valgum, hypotonia, mild cerebral atrophy, osteoporosis, scoliosis	Attention deficit hyperactivity disorder, syndactyly, heart murmur	Congenital hip dysplasia, bilateral sensorineural hearing impairment

References	Ozer et al. (2021)	Jouret et al. (2022)				Bain et al. (2023)		Sun et al. (2025)	Cases in this paper			Total
Reported cases	1	4				2		1	3			23
Age	1 year	23 m	9 years	9 m	12 years	12 years	40 years	4 years	8 years	16^+2^w	16^+3^w	
Gender	F	N/A	N/A	N/A	N/A	F	F	F	F	—	—	
Height	67 cm (−2.29 SD)	6th	<1st	<3rd	<1st	Birth height: 46 cm (z = −1.8)	–	97 cm (<3SD)	N/A	—	—	
Weight	6.4 kg (−3.4 SD)	N/A	N/A	N/A	N/A	Birth weight: 2.64 kg (z = −1.8)	–	12 kg (<3SD)	Birth weight: 1.85 kg (<3SD)	—	—	
Intrauterine/growth disability	+	+	+	+	+	+	–	+	+	—	—	
Psychomotor delay/intellectual disability	+	+	+	+	+	+	+	+	+	—	—	
Microcephaly	+ (38.5 cm (−5.7 SD))	+ (<1st)	+ (<3rd)	+ (<3rd)	+(<3rd)	+ (birth HC: 33 cm (z = −1.9))	+ (<2%)	+ (41.5 cm (−4.7 SD))	+	—	—	
Facial dysmorphism	+	N/A	N/A	+	N/A	+	+	+	+	—	—	
Other features	Attention deficit hyperactivity disorder, thin long fingers with wide fingernails, prominent bilateral sacral dimples, skin eczema, aggressive behavior patterns, positive response to growth hormone therapy	–	Congenital coccygeal sinus	Patent ductus arteriosus, scoliosis	Osteoporosis	Bilateral mild hearing loss, hyperopia	NA	Nephrotic syndrome, mild muscle weakness, congenital heart disease	Precocious puberty, autism spectrum disorder, ankyloglossia	—	—	

N/A: not available.

Among the 20 reported cases, the most prevalent clinical phenotypes were intellectual disability (20/20, 100%), growth retardation (18/20, 90%), and microcephaly (18/20, 90%). Dysmorphic facial features of varying degrees were observed in all 15 cases with sufficient clinical data. Additionally, variable clinical findings included cardiac malformations, congenital hip dysplasia, genitourinary anomalies, osteoporosis, scoliosis, hearing impairment, refractive errors (amblyopia or hyperopia), attention deficit hyperactivity disorder (ADHD), recurrent infections, precocious puberty, and nephrotic syndrome. Given the limited number of reported cases to date, the full clinical spectrum of terminal 19p13.3 pure duplication syndrome remains to be further delineated as more cases are identified.

This study presents the first report of three familial cases of isolated terminal 19p13.3 duplication syndrome arising from a maternal balanced translocation, systematically characterizing its clinical and genetic features. Among these, the proband exhibited classic features of the syndrome (such as intrauterine growth restriction, moderate intellectual disability, microcephaly, and distinctive facial dysmorphism) alongside rare phenotypes, including precocious puberty and autism spectrum disorder. Additionally, this case represents the first documented occurrence of a shortened lingual frenulum in this syndrome, further expanding its clinical phenotypic spectrum. The other two cases were identified via prenatal diagnosis, although level II ultrasound examinations revealed no structural abnormalities, genetic testing elucidated the familial inheritance pattern.

**FIGURE 5:**
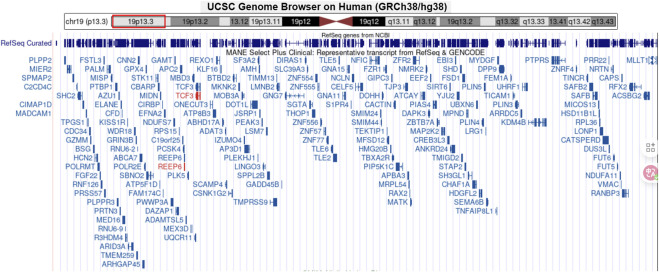
The genomic map of the proband’s terminal pure duplication region on 19p13.3.

Genetic analysis revealed a terminal pure duplication of approximately 6.10 Mb at 19p13.3 (chr19:178,608–6,276,672) in the proband, encompassing 163 OMIM genes such as PIAS4, MAP2K2, CELF5, NFIC, EEF2 (Figure 5). Specifically, PIAS4 is a key candidate gene highly correlated with head circumference; its deletion may result in macrocephaly, while duplication is linked to microcephaly (Tenorio et al., 2020; Özer et al., 2021; Novikova et al., 2017; Nevado et al., 2015). Two fetuses were found to carry duplicated segments at chr19:61,244–6,255,433 (approximately 6.19 Mb) and chr19:61,244–6,271,209 (approximately 6.20 Mb), respectively, their gene content was highly consistent with that of the proband.

The proband in this study was diagnosed with precocious puberty at age 6, representing the second documented case of this phenotype in isolated terminal 19p13.3 duplication syndrome. The first case described by Lybaek et al. (2009) involved an infant with pubertal onset at 5 months of age, presenting with pubic hair growth, breast enlargement, and elevated serum estrogen levels. However, no candidate genes within the duplicated region were definitively linked to the precocious puberty phenotype in that study. The duplicated segment in the present proband encompasses the KISS1R (also known as GPR54) gene, which spans approximately 3.7 kb, encoding the KISS1 receptor, a G protein-coupled receptor. Upon binding to its endogenous ligand kisspeptin, this receptor activates GnRH neurons to secrete gonadotropin-releasing hormone, thereby stimulating the pituitary gland to release luteinizing hormone and follicle-stimulating hormone. This process initiates and maintains the hypothalamic-pituitary-gonadal (HPG) axis, playing a critical regulatory role in pubertal development, sexual maturation and reproductive function. Consequently, KISS1R serves as a vital “molecular switch” for reproductive development. While mutations in this gene are associated with hypogonadotropic hypogonadism and central precocious puberty (Lybaek et al., 2009; Zhang et al., 2023; Cao et al., 2019; Xie et al., 2022; Chen et al., 2024; Zhou and Wu, 2024). However, the pathogenic mechanism underlying its duplication remains unclear. It is hypothesized that KISS1R overexpression may prematurely activate the HPG axis, subsequently triggering central precocious puberty. Thus, the 19p13.3 duplication in the proband, which includes the KISS1R gene, is a strong candidate for the underlying molecular mechanism of the precocious puberty phenotype. This finding provides new genetic evidence linking 19p13.3 microduplication syndrome to central precocious puberty. However, given the current limited number of cases, further clinical and molecular investigations are required to validate the role of KISS1R dosage effects in the pathogenesis of precocious puberty.

Currently reported definitions of the critical region for 19p13.3 microduplication syndrome primarily originate from three perspectives: Orellana et al. proposed a critical region at chr19:3,979,568–4,093,035 (approximately 113.5 kb), encompassing three OMIM genes-PIAS4, ZBTB7A, and MAP2K2 (Nevado et al., 2015); Jouret et al. defined the region as chr19:3,116,922–3,494,377 (approximately 377 kb), encompassing eight protein-coding genes including GNA11, GNA15, and S1PR4 (Jouret et al., 2022); And Sun et al. proposed a critical region at chr19:3,223,850–3,494,377 (approximately 270.5 kb), encompassing the three genes: CELF5, NFIC, and SMIM24 (Sun et al., 2025). However, none of these studies were based exclusively on isolated cases of pure terminal 19p13.3 duplication; Orellana et al. analyzed cases with 19p13.3 microdeletions, while Jouret et al. and Sun et al. incorporated cases with concurrent chromosomal abnormalities. Based on a rigorously selected cohort of isolated terminal 19p13.3 duplications, this study proposes a novel critical region (MOR): chr19:3,223,850–3,536,224, spanning approximately 313 kb. This region encompasses four OMIM genes (CELF5, NFIC, DOHH, FZR1) and two protein-coding genes (SMIM24, SMIM44).

The CELF5 gene (chr19:3,224,661–3,297,076), encoding CUGBP Elav-Like Family Member 5, spans approximately 72.4 kb and encodes an RNA-binding protein. This gene is widely expressed in the central nervous system and muscle tissues, exhibiting peak activity during fetal and infant brain development. CELF5 primarily participates in post-transcriptional regulation, including mRNA splicing, and translation, thereby playing a crucial role in neuronal differentiation, synapse formation, and synaptic plasticity regulation (Gordillo-Sampe et al., 2024; Haque et al., 2025; Huang et al., 2020; Zou et al., 2018; Ladd et al., 2001). Notably, the intellectual disability observed in patients with 19p13.3 microduplication syndrome may be associated with CELF5 overexpression, which potentially disrupts normal neuronal and synaptic development. However, the underlying molecular mechanisms warrant further investigation.

The NFIC gene (chr19:3,359,630–3,469,217), spanning approximately 109.6 kb, belongs to the Nuclear Factor I (NFI) transcription factor family. It encodes a DNA-binding transcription factor that is crucial for tooth, bone, and nervous system development. Its function primarily involves regulating gene expression through DNA binding and protein complex formation. Deletions or mutations in this gene significantly reduce DNA-binding capacity, thereby disrupting the normal transcription of downstream target genes (Jiang et al., 2021; Xu et al., 2022; Sheng et al., 2021; Kim et al., 2015; Wang and Feng, 2017; Zhou et al., 2017; Lee et al., 2020; Steele-Perkins et al., 2003; Pan et al., 2025; Mason et al., 2009). However, the pathogenic mechanism underlying NFIC duplication remains unclear and requires validation through the accumulation of clinical cases and further molecular functional studies.

The DOHH gene (chr19:3,490,824–3,500,674), spanning approximately 9.9 kb in length, encodes deoxyhypusine hydroxylase. This enzyme is essential for activation of eukaryotic translation initiation factor 5A (eIF5A). Given that eIF5A is involved in multiple biological functions, its loss-of-function causes severe multisystem developmental disorders characterized by developmental delay, progressive visual impairment, cardiac structural abnormalities, seizures, and hypotonia (Park et al., 2022; Ziegler et al., 2022; Beltrán-Corbellini et al., 2025; Nakanishi and Cleveland, 2024; Ganapathi et al., 2019). However, the clinical consequences of DOHH gene duplication remain poorly understood. As a potential key driver of 19p13.3 duplication syndrome, DOHH gene overexpression may contribute to developmental delay, intellectual disability, ADHD, and epilepsy. Nevertheless, this association requires further validation through additional clinical cases, and the specific pathogenic mechanisms warrant further investigation.

The FZR1 gene (Fizzy and cell division cycle 20-related protein 1, chr19:3,506,311–3,538,334), also known as HCDH1, spans approximately 32 kb and encodes a key regulator of the cell cycle. It exerts its function primarily by forming a functional complex with the E3 ubiquitin ligase APC/C (anaphase-promoting complex/cyclosome), facilitating mitotic exit into the G1 phase or maintaining quiescence. Beyond its role in somatic cell division, FZR1 plays significant roles in carcinogenesis, neurodevelopment, and reproductive biology. Its dysfunction is associated with embryonic developmental disorders, Alzheimer’s disease, and various tumors (Manivannan et al., 2022; Wan et al., 2017; Ramanujan et al., 2021; Paul et al., 2025; Holt et al., 2011). Current research has established that heterozygous loss-of-function mutations in the FZR1 gene can cause developmental epileptic encephalopathy. However, the pathogenicity of FZR1 duplications has not been definitively reported in the literature. Theoretically, increased copy numbers of this gene may disrupt APC/C-mediated regulatory homeostasis, potentially leading to G1 phase arrest and slowed cell proliferation. These cellular defects may contribute to manifestations such as language delay, growth restriction, and intellectual disability---symptoms consistently observed in 19p13.3 duplication syndrome. Nevertheless, the specific pathogenic mechanism of FZR1 duplication in this syndrome requires further validation through larger clinical cohorts and molecular functional studies.

SMIM24 (chr19:3,473,986–3,480,525) and SMIM44 (chr19:3,482,110–3,483,441) are members of the small integral membrane protein (SMIM) family, both encoding highly conserved transmembrane proteins. SMIM24 is primarily expressed in testes, brain, and immune cells; however, its precise biological function has not yet fully elucidated. It is postulated to participate in cell membrane signaling or protein localization. Research suggests that this protein may be associated with the development of Alzheimer’s disease (Luckett et al., 2023; Lin et al., 2025). To date, no literature reports on the SMIM44 gene have been retrieved from databases such as PubMed, Web of Science Core Collection, and ScienceDirect. As a result, knowledge regarding the biological functions of this gene remains extremely limited. Furthermore, the role of SMIM44 duplication in 19p13.3 microduplication syndrome has yet to be verified through the accumulation of additional clinical cases and the conduct of further experimental studies.

In the familial case reported herein, the terminal 19p13.3 pure duplication in the offspring resulted from a maternal balanced translocation involving the short arm of chromosome 21 and the terminal region of 19p. Typically, terminal 19p13.3 duplications arising from parental balanced translocations are accompanied by concomitant deletions of the partner chromosomal segments. However, in this case, the distal short arm of chromosome 21 and its satellite region primarily comprises clinically insignificant heterochromatin (including the satellite region); thus, its loss did not result in a detectable or clinically relevant deletion. Consequently, the phenotypic manifestation was that of an isolated pure duplication of the 19p13.3 segment. To our knowledge, this represents the first reported family case of a pure terminal duplication at 19p13.3 in offspring arising from a balanced translocation between the short arm of the parental acrocentric chromosome (Group G) and 19p13.3.

## Conclusion

In summary, this study elucidates the genetic mechanism whereby a balanced translocation between the short arm of an acrocentric chromosome and the terminal region of 19p13.3 results in a terminal pure duplication of 19p13.3 in offspring. Furthermore, a new MOR was delineated based on cases with terminal pure 19p13.3 duplications, providing new insights into the associated genotype-phenotype correlations.

## Data Availability

The datasets presented in this study can be found in online repositories. The names of the repository/repositories and accession number(s) can be found in the article/supplementary material.
